# Does a local financial incentive scheme reduce inequalities in the delivery of clinical care in a socially deprived community? A longitudinal data analysis

**DOI:** 10.1186/s12875-015-0279-9

**Published:** 2015-05-14

**Authors:** Liz Glidewell, Robert West, Julia EC Hackett, Paul Carder, Tim Doran, Robbie Foy

**Affiliations:** Leeds Institute of Health Sciences, University of Leeds, Charles Thackrah Building, 101 Clarendon Road, Leeds, UK; Yorkshire and Humber Commissioning Support Unit, Douglas Mill, Bowling Old Lane, Bradford, UK; Department of Health Sciences, University of York, Rowntree Building, York, UK

**Keywords:** Primary health care, Social deprivation, Pay-for-performance

## Abstract

**Background:**

Socioeconomic deprivation is associated with inequalities in health care and outcomes. Despite concerns that the Quality and Outcomes Framework pay-for-performance scheme in the UK would exacerbate inequalities in primary care delivery, gaps closed over time. Local schemes were promoted as a means of improving clinical engagement by addressing local health priorities. We evaluated equity in achievement of target indicators and practice income for one local scheme.

**Methods:**

We undertook a longitudinal survey over four years of routinely recorded clinical data for all 83 primary care practices. Sixteen indicators were developed that covered five local clinical and public health priorities: weight management; alcohol consumption; learning disabilities; osteoporosis; and chlamydia screening. Clinical indicators were logit transformed from a percentage achievement scale and modelled allowing for clustering of repeated measures within practices. This enabled our study of target achievements over time with respect to deprivation. Practice income was also explored.

**Results:**

Higher practice deprivation was associated with poorer performance for five indicators: alcohol use registration (OR 0.97; 95 % confidence interval 0.96,0.99); recorded chlamydia test result (OR 0.97; 0.94,0.99); osteoporosis registration (OR 0.98; 0.97,0.99); registration of repeat prednisolone prescription (OR 0.98; 0.96,0.99); and prednisolone registration with record of dual energy X-ray absorptiometry (DEXA) scan/referral (OR 0.92; 0.86,0.97); practices in deprived areas performed better for one indicator (registration of osteoporotic fragility fracture (OR 1.26; 1.04,1.51). The deprivation-achievement gap widened for one indicator (registered females aged 65–74 with a fracture referred for a DEXA scan; OR 0.97; 0.95,0.99). Two other indicators indicated a similar trend over two years before being withdrawn (registration of fragility fracture and over-75 s with a fragility fracture assessed and treated for osteoporosis risk). For one indicator the deprivation-achievement gap reduced over time (repeat prednisolone prescription (OR 1.01; 1.01,1.01). Larger practices and those serving more affluent areas earned more income per patient than smaller practices and those serving more deprived areas (*t* = −3.99; *p* =0.0001).

**Conclusions:**

Any gaps in achievement between practices were modest but mostly sustained or widened over the duration of the scheme. Given that financial rewards may not reflect the amount of work undertaken by practices serving more deprived patients, future pay-for-performance schemes also need to address fairness of rewards in relation to workload.

## Background

There are widely recognised inequalities in the delivery of primary care, often associated with socioeconomic deprivation [1, 2]. The UK Government made tackling health inequalities an explicit health service priority with the specific aim of improving life expectancy in areas with the worst health and deprivation [3, 4]. The UK Quality and Outcomes Framework (QOF) represents the most substantial initiative within primary care to improve quality and eliminate unacceptable variations in healthcare [5]. It is based upon the premise that financial incentives attached to quality indicators can improve practice and reduce inequalities in healthcare provision. Other healthcare systems have adopted or considered similar initiatives [6–12].

Evidence on the effectiveness of pay-for-performance in primary care is mixed at best [13–15], and is associated with a range of unintended consequences, such as encouraging a ‘tick box’ culture [16], undermining professional autonomy and internal motivation [17], and causing conflict between members of practice staff [18]. In addition, there are concerns that such schemes exacerbated inequalities in the delivery of care; practices serving deprived populations achieved lower levels of performance [19], received less generous financial rewards [20], and potentially inappropriately excluded more patients than those serving more affluent populations [21].

Longitudinal analyses suggest that there were initially gaps in achievement between practices in deprived and affluent areas, but that these closed over time. Doran et al. examined the relationship between socioeconomic inequalities and overall achievement for 48 of the national Quality and Outcomes Framework clinical indicators during the first three years of the scheme’s implementation [22]. National achievement rates increased over the period, and the gap in median achievement narrowed from 4.0 % to 0.8 % between practices in the most and least deprived areas. Ashworth et al. analysed data for blood pressure monitoring and control indicators over the same period, finding that the gaps in achievement rates between practices in affluent and deprived areas had almost closed by the third year of the scheme [23]. A systematic review of largely observational studies concluded there was weak evidence that pay-for-performance reduced inequalities in chronic disease management between socioeconomic groups [24].

The UK Department of Health funded the establishment of a number of locally devolved pay-for-performance schemes in 2008. These offered potential advantages over a national scheme, such as allowing the setting and reward of more ambitious targets than those set nationally, providing opportunities to pilot and improve new quality indicators, and better targeting incentives to reflect local health needs [25–27]. Furthermore, professionals are often reluctant to engage in national quality improvement initiatives because of perceived ineffectiveness or even harm, as well as failures to take account of local contexts [25]. The negotiation of targets with local health professionals may enhance ownership and commitment and strengthen a focus on tackling inequalities [25–27]. These potential gains need to be considered against possible drawbacks, such as increased inequalities in delivery of care if practices in affluent areas can reach targets more easily than those in deprived areas.

The former NHS Primary Care Trust (PCT), NHS Bradford and Airedale, was provided with £3 million funding to develop one such scheme, with a particular emphasis on addressing inequalities in health and the delivery of health care. We examined whether this local pay-for-performance scheme reduced gaps in achievement of targets between the general practices serving the least and most socioeconomically deprived populations. Our accompanying qualitative evaluation explores whether it also avoided unintended consequences [28].

## Methods

### Study design and setting

We undertook a retrospective longitudinal survey of clinical data routinely recorded over four years for the local pay-for-performance scheme. The scheme was introduced in 2007 and ran until 2011, there was no pre-intervention data period. Bradford is the twenty-sixth most deprived out of 326 local authority districts in the UK, with high levels of morbidity [29]. Twenty per cent of its 518,000 people are of South Asian origin as defined by the census [30]. It was not possible to analyse the effect of ethnicity in this study given the unreliability of data coded in primary care.

All 83 practices participated in the evaluation, representing a total population of 555,879 patients. The mean practice IMD for Bradford and Airedale of 37.93 (range 5.6 to 62.3) was higher than the national mean of 22 [31]. Median (range) practice characteristics were as follows: practice list size 6514 (1055–21,374) patients; number of GPs four (0–12); number of GP partners three (0–8); number of salaried GPs one (0–13); number of ‘first fives’ one (0–6). Twenty-three practices had teaching status. The number of practices participating in the local scheme increased over time, with almost all practices contributing to all indicators by the final year (Table 1).

**Table 1:** Number of practices participating in Local QOF by indicator and year

Clinical and public Health Priority	Indicator	2007–08	2008–09	2009-10	2010–11
Weight management	BMI register >25 recorded within 5 years	79	80	80	–
	BMI register >25 recorded last 15 months	79	80	80	–
	BMI >25 intervention last 15 months	79	80	80	–
Alcohol	Register number of units last 27 months	79	80	80	83
	Females >14 units and males >21 units offered brief intervention	79	79	80	83
Learning disabilities	Register over 18 years with a learning disability	77	80	80	83
	Those on register with a review recorded in the preceding 15 months.	77	80	80	83
Osteoporosis	Register of females aged 65–74 with a fracture last 15 months	80	80	80	83
	Proportion of register referred for a DEXA scan	80	80	80	83
	Register aged >16 years who have at least one repeat prescription for oral prednisolone in last 6 months	75	76	80	83
	Percentage on prednisolone register DEXA scan or referral in last 15 months	75	76	80	83
	Percentage over 65 years prescribed prednisolone in the last 6 months, DEXA scan or referral in last 15 months, or assessed for osteoporosis risk.	75	76	80	83
	Register over 75 years had a fragility fracture of the vertebrae, hip, wrist, or humerus since their 75th Birthday	–	–	80	83
	Percentage over 75 years had a fragility fracture of the vertebrae, hip, wrist, or humerus since their 75th Birthday, assessed and treated for Osteoporosis risk ever.	–	–	80	83
Chlamydia screening	Register aged 15 to 24.	42	79	80	–
	Proportion between 15–24 years offered screening and have a recorded result	42	79	80	82

Blank cells indicate where the indicator was not yet introduced or had been withdrawn from the scheme

### The local incentive scheme

Sixteen indicators addressing local clinical and public health priorities were developed, covering five health priorities: weight management; alcohol consumption; learning disabilities; osteoporosis; and chlamydia screening (Table 1). Clinical targets were negotiated between the PCT and local health care professionals and approved by the Local Medical Committee, which represents general practitioners (GPs). Points were awarded on threshold for weight management, alcohol, and osteoporosis indicators (practices were required to achieve the minimum target threshold to score any points, with increasing points awarded for achieving mid and maximum thresholds (Table 2). Each point earned the practice £124.60 in the first year (2007–2008), adjusted for the size of the practice population. Payments for achieving learning disability and chlamydia indicators were awarded on a per patient basis (£50 per patient). Payments, indicators and thresholds were reviewed and refined over the lifetime of the scheme. The PCT provided practices with standardised computerised templates, embedded within electronic patient records, to facilitate recording for both clinical care and indicator production.

**Table 2:** Clinical indicators and payment thresholds

		Trends in practice achievement (%)		Thresholds		
Clinical area	Indicator type	Description	Points (£124.60/point)	Min	Mid	Max
Weight management	Register	OB1 Production of a register of patients aged 16–75 with a BMI ≥25 recorded in the last 5 years	3	No register THEN 0 points	n/a	Have register THEN 3 points
		Omitted 2010-11				
	Register	OB2 Production of a register of patients aged 6–75 with a BMI ≥25 recorded in the last 15 months	7	No registered THEN 0 points	n/a	Have register THEN 7 points
		Omitted 2010-11				
	Process	OB3 Proportion of patients with a BMI ≥25 receiving appropriate intervention in the past 15 months	20	> = 15 % THEN 6 points	> = 30 % THEN 12 points	> = 50 % THEN 20 points
		Omitted 2010-11				
Alcohol	Register	A1 Production of a register of patients aged 16 years and over with a record of the number of units of alcohol consumed on a weekly basis in the past 27 months	10	No register THEN 0 points	n/a	Have register THEN 10 points
	Process	A2 Percentage of patients aged 16 and over who drink >14 units a week (3 units a day) for females and 21 units a week (4 units a day) for males who have been offered a brief intervention	10	IF > = 30 % THEN 3 points	> = 40 % THEN 6 points	> = 50 % THEN 10 points
Learning disabilities	Register	LD1 The practice can produce a register of people over 18 years with a learning disability	£50 per registered patient			
	Process	LD2 Patients over 18 years with LD with a review recorded in the preceding 15 months. Checks include accuracy of prescribed medication, physical health and co-ordination with secondary care	£50 per health check			
Osteoporosis	Register	OST1 Production of a register of female patients aged 65–74 with a fracture in the previous 15 months	2	No register THEN 0 points	n/a	Have register THEN 2 points
		Wording modified in 2010-2011				
	Process	OST 2 Proportion of female patients 65–74 that have had a fracture who have been referred for a dual energy X-ray absorptiometry (DEXA) scan	4	> = 15 % THEN 1 points	> = 30 % THEN 2 points	> = 50 % THEN 4 points
		Wording modified in 2010-2011				
	Register	OST3 Production of a register of male and female patients aged 16 years and over who have received at least one repeat prescription for oral prednisolone in the previous 6 months	2	Not register THEN 0 points	n/a	Have register THEN 2 points
		Wording modified in 2009–10 and 2010-2011				
	Process	OST4 Percentage of patients on register (OST 3) who have a record of a DEXA scan at any time or a referral for a DEXA scan in the previous 15 months	5	> = 15 % THEN 1 points	> = 30 % THEN 3 points	> = 50 % THEN 5 points
		Wording modified in 2010-2011				
	Process	OST5 Percentage of patients aged 65 years and over who have had one or more repeat prescriptions of prednisolone in the last 6 months, who also have a record of a DEXA scan at any time, or a referral for a DEXA scan in the previous 15 months, or have been assessed for osteoporosis risk	5	> = 15 % THEN 1 points	> = 30 % THEN 3 points	> = 50 % THEN 5 points
		Wording modified in 2010-2011				
	Register	OST6 The practice can produce a register of male and female patients aged 75 years and over who have had a fragility fracture of the vertebrae, hip, wrist, or humerus since their 75th Birthday	2	Not Registered THEN 0 points	n/a	Have Registered THEN 2 points
		Introduced 2009–10				
		Wording modified in 2010-2011				
	Process	OST7 Percentage of male and female patients aged 75 years and over who have had a fragility fracture of the vertebrae, hip, wrist, or humerus since their 75th Birthday, who have been assessed and treated for Osteoporosis risk ever.	5	> = 15 % THEN 1 points	> = 30 % THEN 3 points	> = 50 % THEN 5 points
		Introduced 2009–10				
		Wording modified in 2010-2011				
Chlamydia Screening	Register	C1 The practice can produce a register of patients aged 15 to 24 of both sexes	2	Not Registered THEN 0 points	n/a	Have Registered THEN 2 points
		Omitted 2010-11				
	Process	C2 Proportion of patients between 15–24 years old who have been offered screening by their practice and have a recorded test result	£5 per screened patient			

### Data collection and analysis

We invited all 83 primary care practices to opt out of the evaluation if they objected to our analysis of practice-anonymised data. Indicator data were included from April 2007 through March 2011. Registered population socioeconomic deprivation data were extracted from PCT practice profiles, the General Medical Services dataset and the Prescription Pricing Division of the Business Services Authority. Practices were scored using the Indices of Multiple Deprivation (IMD) calculated by the PCT averaging over all patient postcodes registered with the practice (PIMD). IMD measures deprivation across seven domains: income, employment, education, health and disability, crime, barriers to housing and services, and living environment [31]. Deprivation was treated as a continuous variable. Practices in more or less deprived areas were categorised as above or below the median.

A longitudinal analysis adopting a hierarchical multilevel model was undertaken, as performance measures were regarded as nested within practices each year. This enabled use of all the observations in each year, avoided errors of reduced confidence intervals for estimated coefficients, and allowed a comparison of variation between practices and over time. Performance against each indicator was measured as percentage achievement and treated as a continuous measure to describe the proportion of patients achieving a given clinical indicator. We modelled all percentage achievements using a logit transformation (transformed performance = log(Performance/(100-Performance)) to stabilise the variance and improve the validity of the model. Results are presented as regression coefficients. The intra-class correlation coefficient for each indicator fit is provided; lower values show that performance is inconsistent over time. Statistically significant values are expressed as odds ratios. Points assigned for each indicator were available but were not used as any analysis would have had reduced power due to the discretisation of the outcome, and the distribution of the outcome would have been more challenging to model successfully.

Practices’ total financial income was calculated for each year. We derived the income per patient for each of the four financial years by dividing by reported list size. The local incentive payments might be regarded as a weighted basket of indicators where the weights emphasise the importance to the funder. Division by the list size standardised the outcome to a rate per patient registered at each practice. Income as an outcome was modelled using a multilevel regression.

We included the following factors and covariates in the fitted models: financial year; IMD (as a linear term and also as a cubic spline); and modification of IMD by time (i.e. an interaction between IMD and year). List size and total number of GPs were excluded as they were strongly correlated with practice IMD which was the focus of this evaluation.

For practice income a full model was initially fitted and stepping down was implemented. We included the following factors and covariates in the fitted models: list size (as a linear term and also as a cubic spline); financial year; total number of GPs, number of partners, number of salaried GPs; number of ‘first fives’ (i.e. recently qualified GPs); practice teaching status (i.e. whether involved in training of GPs) and IMD (as a linear term and also as a cubic spline); and modification of IMD by time (i.e. an interaction between IMD and year). List size was included as learning disability and chlamydia screening indicators were proportional to list size and activity level. Covariates or factors were dropped if the effect size was small and statistical significance did not reach the 10 % significance level. We aimed to achieve sufficiently parsimonious models for which coefficients could be estimated efficiently. All analysis was undertaken using the R statistical package version 2.12.0 [32] and the nlme package version 3.1 [33]. The statistical interaction term was included to assess the impact of the local incentive on health inequalities and estimate if any gap has widened or narrowed through the duration of the scheme.

### Ethical review

The study was approved by National Research Ethics Service East Midlands- Nottingham 2 Committee (11/EM/0184).

## Results

### Analysis of achievement

Table 3 shows levels of population coverage achieved for each indicator. Against backgrounds of overall increasing achievement, there was still substantial scope for improvement towards the end of the scheme. For example, just over a quarter (26.1 %) of the population had a recorded BMI within the preceding 15 months by 2009–10 whilst only 6.1 % of patients aged 15–24 years had recorded chlamydia screening test results by 2010–11.

**Table 3:** Achievement on clinical process and outcome indicators over time^a^

Clinical Area	Indicator	Achievement	Year 1 2007-08	Year 2 2008-09	Year 3 2009-10	Year 4 2010-11
Weight management	BMI register >25 recorded within 5 years	Mean percentage achievement (SD)	32.7 (7.7)	36.4 (7.4)	38.7 (9.5)	NA
		Mean practices in more deprived areas	31.2 (8.2)	34.9 (7.6)	36.7 (11.2)	(−)
		Mean practices in less deprived areas	34.2 (7.0)	38.0 (6.9)	40.7 (7.0)	(−)
	BMI register >25 recorded last 15 months	Mean percentage achievement (SD)	19.9 (6.1)	24.5 (6.7)	26.1 (8.0)	NA
		Mean practices in more deprived areas	19.4 (6.0)	23.6 (6.8)	26.1 (9.4)	(−)
		Mean practices in less deprived areas	20.5 (6.1)	25.4 (6.5)	26.1 (6.5)	(−)
	BMI >25 intervention last 15 months	Mean percentage achievement (SD)	21.1 (17.8)	61.1 (23.4)	57.4 (18.8)	NA
		Mean practices in more deprived areas	17.0 (15.2)	59.7 (23.6)	54.5 (21.1)	(−)
		Mean practices in less deprived areas	25.3 (19.4)	62.6 (23.5)	60.2 (16.0)	(−)
Alcohol	Register number of units last 27 months	Mean percentage achievement (SD)	19.5 (14.8)	32.1 (17.2)	44.4 (20.9)	54.8 (20.4)
		Mean practices in more deprived areas	13.1 (10.8)	23.8 (15.8)	39.4 (24.9)	54.9 (23.5)
		Mean practices in less deprived areas	26.1 (15.6)	40.4 (14.5)	49.5 (14.5)	54.7 (16.6)
	Females >14 units and males >21 units offered brief intervention	Mean percentage achievement (SD)	33.9 (28.5)	69.9 (23.4)	74.7 (24.2)	74.8 (20.7)
		Mean practices in more deprived areas	26.4 (26.6)	70.6 (26.2)	71.8 (29.7)	71.0 (23.3)
		Mean practices in less deprived areas	41.7 (28.6)	69.2 (20.4)	77.5 (17.0)	79.0 (16.8)
Learning disabilities	Register over 18 years with a learning disability	Mean percentage achievement (SD)	.3 (.2)	.4 (.3)	.4 (.2)	.5 (.3)
		Mean practices in more deprived areas	0.28 (0.21)	0.33 (0.26)	0.34 (0.26)	0.51 (0.29)
		Mean practices in less deprived areas	0.29 (0.23)	0.40 (0.24)	0.40 (0.23)	0.50 (0.27)
	Those on register with a review recorded in the preceding 15 months.	Mean percentage achievement (SD)	.9 (7.0)	54.2 (37.1)	59.2 (34.0)	58.8 (33.5)
		Mean practices in more deprived areas	0.2 (1.2)	48.6 (38.4)	49.4 (37.9)	52.7 (36.7)
		Mean practices in less deprived areas	1.6 (9.7)	59.7 (35.3)	69.0 (26.5)	65.4 (28.5)
Osteoporosis	Register of females aged 65–74 with a fracture last 15 months	Mean percentage achievement (SD)	.6 (.8)	1.0 (1.0)	1.3 (1.1)	.6 (.5)
		Mean practices in more deprived areas	0.57 (0.89)	0.80 (1.14)	1.19 (1.44)	0.45 (0.52)
		Mean practices in less deprived areas	0.66 (0.60)	1.17 (0.91)	1.48 (0.73)	0.72 (0.40)
	Proportion of register referred for a DEXA scan	Mean percentage achievement (SD)	1.1 (6.3)	46.1 (45.1)	51.1 (42.4)	42.0 (39.2)
		Mean practices in more deprived areas	1.3 (7.9)	32.0 (45.5)	39.0 (46.2)	29.0 (40.4)
		Mean practices in less deprived areas	0.9 (4.2)	60.1 (40.7)	63.3 (34.7)	57.8 (32.2)
	Register aged >16 years who have at least one repeat prescription for oral prednisolone in last 6 months	Mean percentage achievement (SD)	.9 (1.3)	.4 (.5)	.2 (.2)	.3 (1.1)
		Mean practices in more deprived areas	0.31 (0.32)	0.29 (0.34)	0.21 (0.19)	0.23 (0.26)
		Mean practices in less deprived areas	0.90 (1.72)	0.54 (0.57)	0.22 (0.15)	0.45 (1.55)
	Percentage on prednisolone register DEXA scan or referral in last 15 months	Mean percentage achievement (SD)	16.6 (25.9)	65.0 (39.1)	58.0 (38.5)	66.3 (36.6)
		Mean practices in more deprived areas	8.4 (17.3)	58.0 (40.8)	46.1 (41.3)	51.3 (42.0)
		Mean practices in less deprived areas	24.2 (30.2)	71.3 (37.0)	69.9 (31.8)	82.5 (20.0)
	Percentage over 65 years prescribed prednisolone in the last 6 months, DEXA scan or referral in last 15 months, or assessed for osteoporosis risk.	Mean percentage achievement (SD)	31.0 (34.4)	66.1 (39.4)	60.6 (41.4)	1.8 (3.2)
		Mean practices in more deprived areas	31.4 (38.7)	56.3 (45.5)	49.6 (47.3)	1.1 (1.4)
		Mean practices in less deprived areas	30.6 (30.3)	74.9 (30.9)	71.6 (31.3)	2.5 (4.3)
	Register over 75 years had a fragility fracture of the vertebrae, hip, wrist, or humerus since their 75th Birthday	Mean percentage achievement (SD)	NA	NA	1.1 (.9)	60.7 (43.5)
		Mean practices in more deprived areas	– (−)	– (−)	0.7 (0.7)	36.5 (45.3)
		Mean practices in less deprived areas	– (−)	– (−)	1.4 (0.9)	86.6 (20.6)
	Percentage over 75 years had a fragility fracture of the vertebrae, hip, wrist, or humerus since their 75th Birthday, assessed and treated for Osteoporosis risk ever.	Mean percentage achievement (SD)	NA	NA	4.7 (2.8)	56.3 (39.5)
		Mean practices in more deprived areas	– (−)	– (−)	3.4 (2.7)	36.8 (41.0)
		Mean practices in less deprived areas	– (−)	– (−)	6.2 (2.0)	77.2 (24.7)
Chlamydia Screening	Register aged 15 to 24.	Mean percentage achievement (SD)	100 (0)	100 (0)	100 (0)	NA
		Mean practices in more deprived areas	100 (0)	100 (0)	100 (0)	(−)
		Mean practices in less deprived areas	100 (0)	100 (0)	100 (0)	(−)
	Proportion between 15–24 years offered screening and have a recorded result	Mean percentage achievement (SD)	1.7 (1.7)	5.7 (4.9)	6.1 (4.9)	6.1 (7.5)
		Mean practices in more deprived areas	1.53 (1.43)	4.45 (4.80)	6.10 (5.03)	6.61 (9.78)
		Mean practices in less deprived areas	1.72 (1.87)	6.98 (4.67)	7.16 (4.43)	5.63 (3.77)

^a^Blank cells indicate where the indicator was not yet introduced or had been withdrawn from the scheme

Table 4 provides the coefficients in the regression for each indicator. In Table 5 we present statistically significant effects as odds ratios. We found that higher practice deprivation was associated with poorer achievement for five indicators over a four-year incentivised period: alcohol use registration (OR 0.97; 95 % confidence interval 0.96,0.99; *p* = 0.006); recorded chlamydia test result (OR 0.97; 0.94,0.99; *p* = 0.002); osteoporosis registration (OR 0.98; 0.97,0.99; *p* = 0.010); registration of repeat prednisolone prescription (OR 0.98; 0.96,0.99; *p* = 0.001); and prednisolone registration with record of dual energy X-ray absorptiometry (DEXA) scan or referral (OR 0.92; 0.86,0.97; *p* = 0.003). Higher practice deprivation was associated with better achievement for one indicator, registration of osteoporotic fragility fracture (OR 1.26; 1.04,1.51; *p* = 0.016). The deprivation-achievement gap widened over the four years of the scheme for one indicator, registered females aged 65–74 with a fracture referred for a DEXA scan (OR 0.97; 0.95,0.99; *p* = 0.028). Two other indicators that were introduced in the last two years of the scheme followed a similar trend: registration of fragility fracture (OR 0.88; 0.82, 0.94; *p* = 0.001); and over-75’s with a fragility fracture assessed and treated for osteoporosis risk (OR 0.92; 0.87, 0.97; *p* = 0.003). For one indicator, registration of repeat prednisolone prescription, the deprivation-achievement gap closed over time (OR 1.01; 1.01, 1.01; *p* = 0.001).

**Table 4:** Performance on clinical indicators over 2007–11^a^

Clinical and public Health Priority	Indicator	2007–2008	2008–2009	2009-2010	2010–2011	PIMD	PIMD*Year	ICC
Weight management	BMI register >25 recorded within 5 years	−0.74 (−1.11,-0.38)	−0.57 (−0.93,-0.20)	−0.58 (−0.94,-0.22)	–	−0.000 (−0.009,0.009)	−0.005 (−0.011,0.000)	0.35
	BMI register >25 recorded last 15 months	−1.51 (−1.86,-1.15)	−1.23 (−1.59,-0.88)	−1.26 (−1.61,-0.90)	–	0.002 (−0.007,0.011)	−0.004 (−0.009,0.001)	0.41
	BMI >25 intervention last 15 months	−1.32 (−2.06,-0.58)	1.10 (0.36,1.83)	0.50 (−0.23,1.22)	–	−0.008 (−0.026,0.010)	−0.004 (−0.018,0.010)	0.04
Alcohol	Register number of units last 27 months	−0.87 (−1.48,-0.25)	0.02 (−0.59,0.64)	0.54 (−0.08,1.16)	1.16 (0.55,3.20)	−0.027 (−0.042,-0.011)	0.006 (−0.000,0.012)	0.38
	Females >14 units and males >21 units offered brief intervention	−0.71 (−1.92,0.51)	2.06 (0.85,3.28)	2.09 (0.88,3.30)	2.37 (1.16,3.58)	−0.019 (−0.048,0.011)	0.008 (−0.006,0.022)	0.13
Learning disabilities	Register over 18 years with a learning disability	−6.05 (−6.41,-5.69)	−5.80 (−6.16,-5.44)	−5.75 (−5.78,-5.39)	−5.42 (−5.78,-5.06)	−0.001 (−0.010,0.008)	0.002 (−0.000,0.003)	0.80
	Those on register with a review recorded in the preceding 15 months.	−5.64 (−7.35,-3.94)	0.84 (−0.87,2.54)	1.42 (−0.29,3.13)	1.54 (−0.16,3.25)	−0.033 (−0.075,0.010)	−0.008 (−0.025,0.009)	0.33
Osteoporosis	Register of females aged 65–74 with a fracture last 15 months	−4.96 (−5.53,-4.39)	−4.54 (−5.11,-3.97)	−4.16 (−4.73,-3.59)	−4.78 (−5.34,-4.21)	−0.019 (−0.033,-0.004)	−0.004 (−0.010,0.003)	0.20
	Proportion of register referred for a DEXA scan	−5.14 (−7.50,-2.78)	0.74 (−1.62,3.10)	1.42 (−0.94,3.78)	0.43 (−1.92,2.79)	−0.043 (−0.102,0.015)	−0.030 (−0.056,-0.003)	0.21
	Register aged >16 years who have at least one repeat prescription for oral prednisolone in last 6 months	−4.80 (−5.16,-4.44)	−4.97 (−5.33,-4.60)	−5.45 (−5.81,-5.08)	−5.35 (−5.72,-4.99)	−0.023 (−0.032,-0.014)	0.007 (0.004,0.010)	0.59
	Percentage on prednisolone register DEXA scan or referral in last 15 months	−1.10 (−3.40,1.19)	4.66 (2.36,6.97)	4.09 (1.77,6.41)	4.85 (2.54,7.17)	−0.089 (−0.147,-0.030)	−0.002 (−0.026,0.022)	0.33
	Percentage over 65 years prescribed prednisolone in the last 6 months, DEXA scan or referral in last 15 months, or assessed for osteoporosis risk.	−0.51 (−2.69,1.67)	2.99 (0.81,5.18)	2.73 (0.53,4.93)	−2.95 (−5.14,-0.75)	−0.047 (−0.102,0.009)	−0.008 (−0.031,0.014)	0.32
	Register over 75 years had a fragility fracture of the vertebrae, hip, wrist, or humerus since their 75th Birthday	–	–	−13.27 (−20.11,-6.43)	−7.39 (−14.24,-0.55)	0.228 (0.042,1.256)	−0.131 (−0.203,-0.059)	0.08
	Percentage over 75 years had a fragility fracture of the vertebrae, hip, wrist, or humerus since their 75th Birthday, assessed and treated for Osteoporosis risk ever.	–	–	−8.01 (−13.14,-2.87)	−4.53 (−9.67,0.60)	0.124 (−0.014,0.262)	−0.082 (−0.136,-0.028)	0.22
Chlamydia screening	Register aged 15 to 24.	All participating practices record 100 % for 07–08, 08–09, and 09–10.						
	Proportion between 15–24 years offered screening and have a recorded result	−4.00 (−4.80,-3.21)	−2.15 (−2.98,-1.33)	−2.11 (−2.93,-1.29)	−2.28 (−3.10,-1.46)	−0.033 (−0.054,-0.012)	0.002 (−0.004,0.008)	0.69

^a^Blank cells indicate where the indicator was not yet introduced or had been withdrawn from the scheme

**Table 5:** Multilevel modelling accounting for clustering of years within practices^a^

Clinical area	Description	OR Practice IMD per deprivation point	P	OR Practice IMD per deprivation point per year	P
Weight management	BMI register >25 recorded within 5 years				
	BMI register >25 recorded last 15 months				
	BMI >25 intervention last 15 months				
Alcohol	Register number of units last 27 months	0.974 (0.959,0.989)	0.006		
	Females >14 units and males >21 units offered brief intervention				
Learning disabilities	Register over 18 years with a learning disability				
	Those on register with a review recorded in the preceding 15 months.				
Osteoporosis	Register of females aged 65–74 with a fracture last 15 months	0.982 (0.968,0.996)	0.010		
	Proportion of register referred for a DEXA scan			0.971 (0.946,0.997)	0.028
	Register aged >16 years who have at least one repeat prescription for oral prednisolone in last 6 months	0.977 (0.969,0.986)	0.001	1.007 (1.004,1.010)	0.001
	Percentage on prednisolone register DEXA scan or referral in last 15 months	0.915 (0.864,0.970)	0.003		
	Percentage over 65 years prescribed prednisolone in the last 6 months, DEXA scan or referral in last 15 months, or assessed for osteoporosis risk.				
	Register over 75 years had a fragility fracture of the vertebrae, hip, wrist, or humerus since their 75th Birthday	1.256 (1.043,1.511)	0.016	0.877 (0.816,0.943)	0.001
	Percentage over 75 years had a fragility fracture of the vertebrae, hip, wrist, or humerus since their 75th Birthday, assessed and treated for Osteoporosis risk ever.			0.921 (0.873,0.972)	0.003
Chlamydia Screening	Register aged 15–24.				
	Proportion between 15–24 years offered screening and have a recorded result	0.967 (0.947,0.988)	0.002		

^a^Only statistically significant odds ratios are presented

### Income analysis

**Fig. 1:**
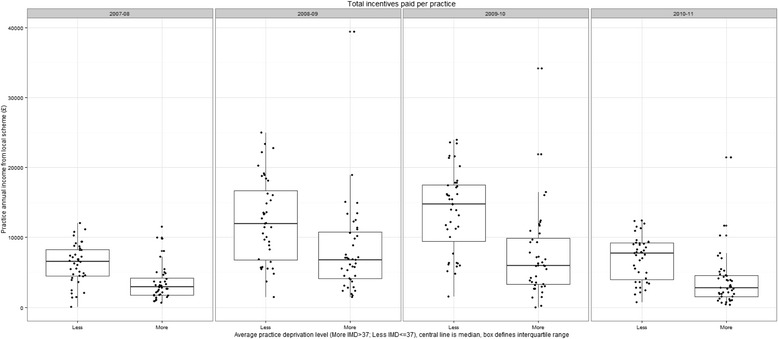
Boxplot of distribution of annual practice income for practices in more and less deprived areas by financial year

Fig. 1 shows the distribution of total practice income for practices in more and less deprived areas by year. The total funding available also reflected the trajectory for the median values, with twice as much funding available in the two middle years.

Hierarchical multiple regression indicated that income over time was positively correlated with practice size (coefficient 0.0000161; *t* = 2.27; *p* = 0.026) and negatively correlated with IMD (coefficient −0.00664; *t* = −3.99; *p* = 0.0001; Table 6). For convenience of interpretation, we report size and practice IMD ‘centred’ around the average median values. No intercept was fitted so that the coefficients for each financial year represent the average payment per patient for that year.

**Table 6:** Regression of practice income levels

Factor	Coefficient	SE	DF	*t*-value	*p*-value
2007–08	0.697	0.035	237		
2008–09	1.466	0.035	237		
2009–10	1.488	0.035	237		
20019–11	0.762	0.035	237		
List-size-6643	0.000016	0.000008	81	2.27	0.0257
IMD-36	−0.00664	0.00166	81	−3.99	0.0001

Note: The model was fitted to 323 income values grouped within 83 practices

Factors such as the number of GPs, partners, number of ‘first fives,’ and teaching practice status appeared well represented in the model by list size. The association with list size was well represented by a simple linear term. The coefficient reported is per patient. Thus an increase in list size by 6000 patients is associated with an additional £0.10 per patient. There was an association with deprivation as measured by the average practice IMD (PIMD) score for a practice. An increase in PIMD by 15 points (one standard deviation) was associated with a reduction in payment of £0.10 per patient; practices serving more deprived populations receive relatively lower payments. A modification to the deprivation term was considered, but found to be very small and far from statistical significance. In consequence, the model predicted neither widening nor narrowing of inequality in practice income.

Larger practices and those serving more affluent areas earned more income per patient than smaller practices and those serving more deprived areas (t −3.99 *p* = 0.0001). A practice of average list size (6719 patients) and average deprivation (PIMD = 36.51) would earn around £7500 per year; a practice with 3598 more patients (one standard deviation in list size) would earn £11,962 (£4462 more). A practice with an additional deprivation of one standard deviation 15.34) would earn £7081 (£419 less).

## Discussion

We found that practice level deprivation was associated with lower achievement for five out of 16 indicators in a local primary care pay-for-performance scheme. Deprivation-achievement gaps closed over time for one indicator but opened up for three others. Overall, the observed differences were modest. Almost all of the indicators we analysed involved cumulative registration of at-risk patient populations, so that we would have expected gaps to close over time once ‘ceilings’ in achievement had been reached. For the most part, this did not occur. We also found that considerable scope for improvement still existed by the end of the scheme. For example, the final mean proportions of targeted patients with recorded alcohol use and offers of screening and recording of chlamydia test results were 55 and 6.2 % respectively. This is the first inequalities analysis of a local pay-for-performance scheme; our findings on achievement contrast with those of the national scheme [24]. We also found inequalities in income with smaller practices serving more deprived populations earning less than larger practices serving more affluent populations. This is in line with analyses for the national scheme that found an inconsistent link between workload and reward for practices in deprived areas, suggesting that smaller practices serving more deprived populations had to work harder to earn the same amount per patient [20]. Our accompanying qualitative study suggested that financial reward was the main extrinsic driver for smaller practices serving more deprived populations. However there were concerns that financial rewards from the local scheme may not have been worth the effort involved in achieving targets. The national scheme was perceived as more important as there was more remuneration attached [28].

There were four main study limitations. First, we only examined one local pay-for-performance scheme in a relatively deprived population; we cannot assume that our findings would be generalizable elsewhere or to schemes with different indicators and targets. Second, as with the national Quality and Outcomes Framework, the content of indicators and the thresholds for levels of achievement both changed year upon year and some were withdrawn. Although this is challenging for researchers and complicates the interpretation of trends, our evaluation subsequently reflected ‘real world’ circumstances. Outcomes were not standardised by the number of patients with each condition as many of the targets involved establishing a register. List size (strongly correlated with practice deprivation) and ratio of GPs to patients were not included as covariates (no data on number of sessions worked). Third, as Alshamsan et al. noted for similar analyses, the absence of any pre-intervention data means that we cannot attribute any reductions in inequalities to the scheme [24]. Fourth, deprivation was summarised at the level of lowest super-output areas, which may contain areas with different levels of deprivation. In addition, deprivation scores were assigned on the basis of averaging over the patient list postcodes, potentially resulting in an underestimation of the association between patient-level socioeconomic deprivation and achievement.

Although all practices had signed up to the scheme by its final year, the overall levels of achieved population coverage were limited; practices may have reduced effort once the relatively modest targets had been met. Our accompanying qualitative evaluation suggested no particularly strong sense of local ownership of the scheme, or its public health-oriented indicators, which could have motivated stronger efforts compared with the national scheme [28]. The national QOF scheme is worth around £130,000 in total per annum to the average practice whereas the local scheme is worth £7500, translating into around £970 and £470 per indicator respectively [34]. The income per point was similar in both schemes but practices may have focused more attention on the larger overall income available from the national QOF.

Larger practices serving more affluent populations are often better positioned, in terms of having greater capacity to scale up and industrialise activity, for better and earlier achievement than their counterparts. Whilst there would be risks in being seen to penalise the former, local policy-makers need to generate a consensus on ‘fairness’ and the need for quality improvement initiatives that proportionately compensate practices serving more deprived patient populations.

With continuing reforms in English primary care at least rhetorically geared towards promoting local autonomy [25–27], the number and range of local incentivised schemes are likely to grow. Given the cost of implementing local schemes there is an urgent need for further evaluations of both effectiveness and equity which improve upon our own, which was limited by the lack of pre-intervention data and relatively small number of indicators.

## Conclusion

Practice level deprivation was associated with modestly lower achievement for around a third of 16 indicators in a local primary care pay-for-performance scheme. There was relatively little change over time, with deprivation-achievement gaps closing for one indicator but opening up for three others. Furthermore, given that inequalities in financial reward may not reflect the amount of work undertaken by smaller practices serving more deprived patients given the fixed costs of setting up and managing disease registers, future iterations and evaluations of local pay-for-performance schemes need to address fairness of rewards according to effort as well as effectiveness.

## Abbreviations

QOF: Quality and outcomes framework
UK: United Kingdom
PCT: Primary care trust
GP: General practitioner
IMD: Index of multiple deprivation
SD: Standard deviation
OR: Odds ratio
PIMD: Primary care trust assigned average index of multiple deprivation based on patient postcodes
ICC: Intra cluster coefficient
